# Stereotactic aspiration combined with gamma knife radiosurgery for the treatment of cystic brainstem metastasis originating from lung adenosquamous carcinoma: A case report

**DOI:** 10.3892/ol.2015.2968

**Published:** 2015-02-16

**Authors:** CHAO DU, ZHAOHUI LI, ZHIJIA WANG, LIPING WANG, YU TIAN

**Affiliations:** 1 Department of Neurosurgery, China-Japan Union Hospital of Jilin University, Changchun, Jilin 130033, P.R. China; 2 Department of Radiology, China-Japan Union Hospital of Jilin University, Changchun, Jilin 130033, P.R. China; 3 Department of Pathology, China-Japan Union Hospital of Jilin University, Changchun, Jilin 130033, P.R. China

**Keywords:** adenosquamous carcinoma, gamma knife radiosurgery, brainstem metastasis, stereotactic aspiration

## Abstract

Brainstem metastases have a poor prognosis and are difficult to manage. The present study describes the first case of histopathologically-confirmed brainstem metastasis originating from lung adenosquamous carcinoma, and discusses the outcomes of treatment by stereotactic aspiration combined with gamma knife radiosurgery (GKRS). A 59-year-old female presented with a cystic mass (15×12×13 mm; volume, 1.3 cm^3^) located in the pons, two years following surgical treatment for adenosquamous carcinoma of the lung. The patient received initial GKRS for the lesion in the pons with a total dose of 54.0 Gy, however, the volume of the mass subsequently increased to 3.9 cm^3^ over a period of three months. Computed tomography-guided stereotactic biopsy and aspiration of the intratumoral cyst were performed, yielding 2.0 cm^3^ of yellow-white fluid. Histology confirmed the diagnosis of adenosquamous carcinoma. Aspiration provided immediate symptomatic relief, and was followed one week later by repeat GKRS with a dose of 12.0 Gy. The patient survived for 12 months following the repeat GKRS; however, later succumbed to the disease after lapsing into a two-week coma. The findings of this case suggest that stereotactic aspiration of cysts may improve the effects of GKRS for the treatment of cystic brainstem metastasis; the decrease in tumor volume allowed a higher radiation dose to be administered with a lower risk of radiation-induced side effects. Therefore, stereotactic aspiration combined with GKRS may be an effective treatment for brainstem metastasis originating from adenosquamous carcinoma.

## Introduction

Brain metastases are common intracranial malignancies, occurring in 15–40% of adult cancer patients. Only 3–5% of all brain metastases are located in the brainstem. The primary tumor with the highest rate of brainstem involvement is lung cancer, followed by breast cancer, ovarian cancer, colorectal cancer, renal cell carcinoma and melanoma. Brainstem metastases have a poor prognosis and are particularly challenging to manage; clinical reviews of patients treated for brainstem metastases have reported median survival times ranging from 1–11 months (1–12). Surgical resection is rarely a viable option due to the high risk of developing new neurological deficits or worsening of existing deficits as the majority of drugs which penetrate the blood brain barrier have been found to exhibit low efficacy in this context (5–7). Chemotherapy is also of limited use in brainstem metastases. Stereotactic radiosurgery (SRS) procedures, including gamma knife radiosurgery (GKRS) and linear accelerator (LINAC), have proven to be effective treatment modalities for brainstem metastases (2–21). To the best of our knowledge, the current study reports the first patient with histopathologically-confirmed brainstem metastasis originating from lung adenosquamous carcinoma, and discusses the outcome of stereotactic aspiration and GKRS. Written informed consent was obtained from the patient’s family.

## Case report

**Figure 1:**
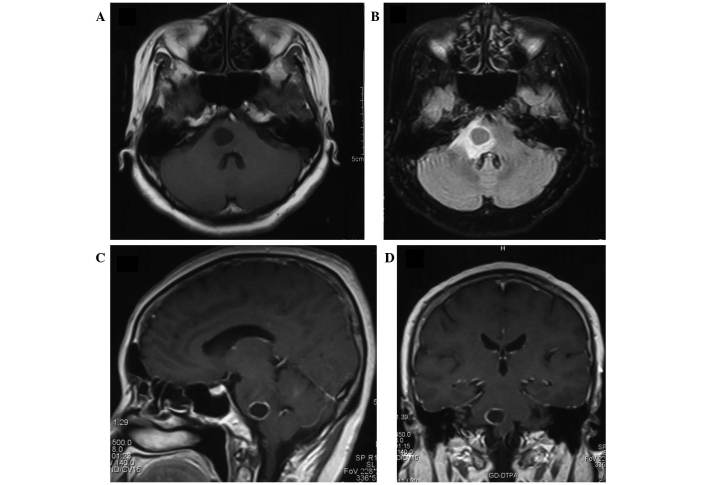
Magnetic resonance images of the cystic brainstem metastasis taken one week after the onset of symptoms. The tumor was 15×12×13 mm in size (volume, 1.3 cm^3^) prior to the initial gamma knife radiosurgery. (A) Axial T1-weighted image; (B) axial T2-weighted, fluid-attenuated inversion recovery image; (C) sagittal contrast-enhanced T1-weighted image; and (D) coronal contrast-enhanced T1-weighted image. FoV, field of view; GD-TPA, gadolinium diethylenetriamine pentaacetic acid.

**Figure 2:**
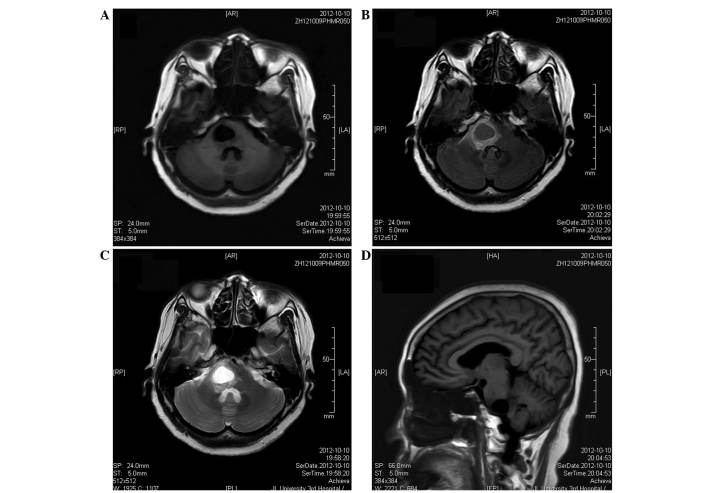
Magnetic resonance images obtained three months following the initial gamma knife radiosurgery. The cystic mass increased in size to 22×17×19 mm (volume: 3.9 cm^3^). (A) Axial T1-weighted image; (B) axial T2-weighted, fluid-attenuated inversion recovery image; (C) axial diffusion-weighted image; and (D) sagittal T1-weighted image.

A 59-year-old Chinese female presented to the Department of Neurosurgery, The Affiliated Hospital of Beihua University (Jilin, China) with a two-week history of vertigo and gait instability on May 19, 2012. Magnetic resonance imaging (MRI) revealed a cystic mass located in the pons. The cystic brainstem tumor initially measured 15×12×13 mm (volume, 1.3 cm^3^), and the cyst wall exhibited annular enhancement (Fig. 1). The patient had undergone surgical treatment for primary lung cancer, pathologically diagnosed as adenosquamous carcinoma, at Jilin Province Cancer Hospital (Changchun, China) two years prior to the current presentation. Brainstem metastases were diagnosed based on the clinical and neuroimaging findings, and the patient received initial GKRS for the lesion in the pons over 40 days. The total radiosurgical dose applied to the tumor was 54.0 Gy in 2 Gy daily fractions. MRI, three months following the completion of GKRS, revealed that the cystic mass had increased in size to 22×17×19 mm (volume, 3.9 cm^3^) (Fig. 2). The patient was subsequently referred to the Department of Neurosurgery, China-Japan Union Hospital of Jilin University (Changchun, China) for further treatment.

**Figure 3:**
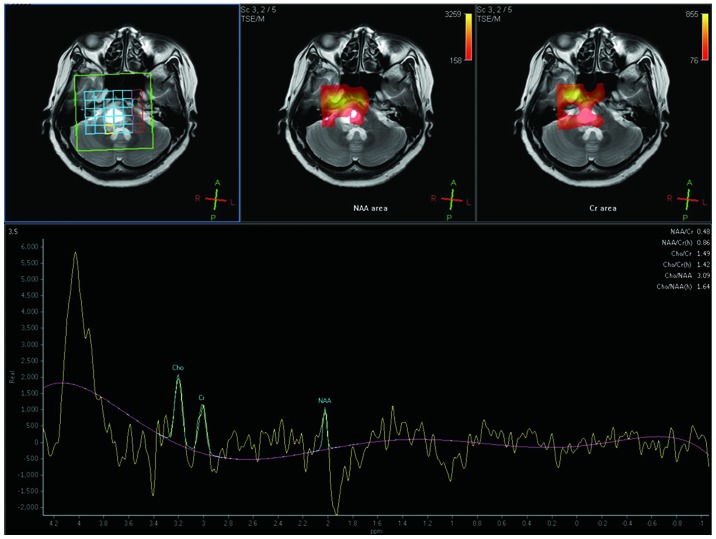
Images from magnetic resonance spectroscopy obtained three months following the initial gamma knife radiosurgery. The yellow shading indicates peritumoral edema. The choline/N-acetylaspartate ratio was 3.09. A, anterior; Cho, choline; Cr, creatine; L, left; NAA, N-acetylaspartate; P, posterior; PPM, parts per million; R, right.

**Figure 4:**
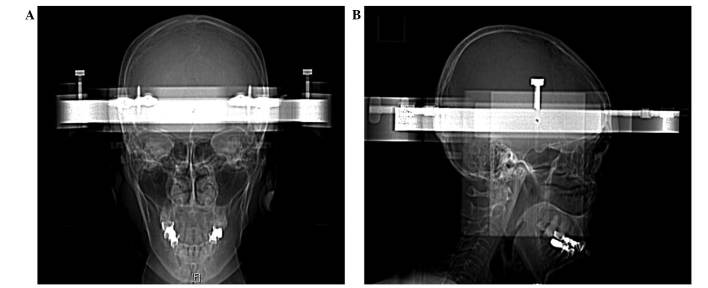
Computed tomographic images showing the setup for stereotactic aspiration. (A) Antero-posterior view; (B) lateral view.

**Figure 5:**
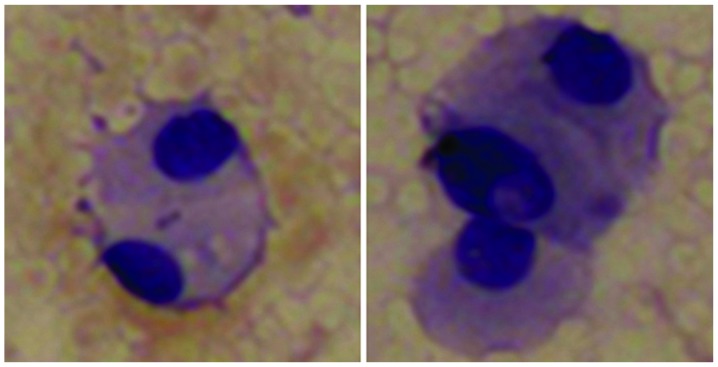
Smear of the cystic fluid showing atypical cells.

**Figure 6:**
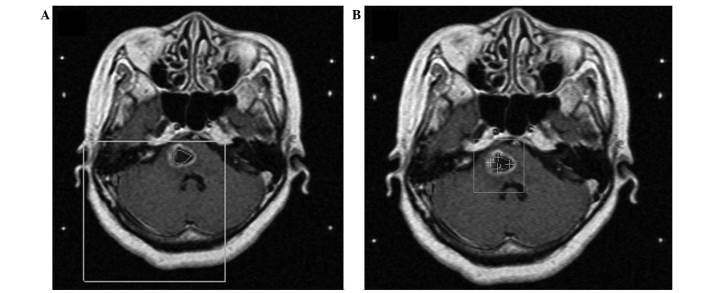
Magnetic resonance images obtained one week following stereotactic aspiration surgery. The tumor decreased in size to 18×15×14 mm (volume, 1.9 cm^3^). (A) Axial T1-weighted images. (B) Axial T1-weighted images showing dose planning of the tumor for gamma knife radiosurgery.

**Figure 7:**
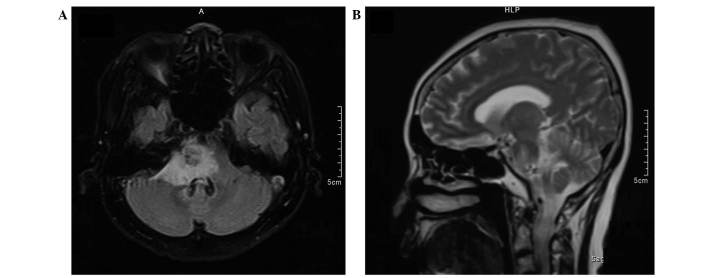
Magnetic resonance images obtained five months following stereotactic aspiration. The tumor size was 12×13×14 mm (volume, 1.2 cm^3^). New metastases are visible in the cerebellum. (A) Axial T1-weighted, fluid-attenuated inversion recovery image and (B) sagittal T2-weighted image.

Upon admission, magnetic resonance spectroscopy (MRS) with peritumoral measurements revealed that the choline/N-acetylaspartate (Cho/NAA) ratio was 3.09 (Fig. 3). Computed tomography-guided stereotactic biopsy and aspiration of the intratumoral cyst, performed three months following the initial GKRS, yielded 2.0 cm^3^ of yellow-white fluid (Fig. 4), and a smear of the hydatid fluid showed atypical cells (Fig. 5). Histopathology revealed the biopsy specimen to be a metastatic adenosquamous carcinoma originating from the lung. The sample exhibited similar hematoxylin and eosin morphology to lung adenosquamous carcinoma with predominant well-differentiated adenocarcinoma associated with heterologous elements of squamous cell carcinoma. An MRI scan for gamma knife radiosurgery planning conducted following stereotactic aspiration showed that the cystic tumor in the pons had decreased in size to 18×15×14 mm (volume, 1.9 cm^3^; Figure 6). The patient’s vertigo and gait instability improved within three days following the aspiration, and a second GKRS was performed one week later (dose, 12.0 Gy). Following discharge from hospital, the patient was alert and the neurological symptoms had resolved. A further MRI scan was performed five months subsequent to the repeat GKRS, revealing that the brainstem metastasis had reduced to 12×13×14 mm (volume, 1.2 cm^3^), without severe radiation-induced edema (Fig. 7). MRI also indicated that the brainstem metastasis was well controlled. The patient survived for 12 months following the repeat GKRS, however, later succumbed to the disease after lapsing into a two-week coma following the development and progression of new brain metastases.

## Discussion

A number of reports have described the treatment of brainstem metastasis, including pontine metastasis (1–11). The most common site of primary malignancy in patients with metastases to the brainstem is the lungs; the majority of such malignancies are adenocarcinomas (6,7,9,10) and, less commonly, squamous cell carcinomas (8). SRS is the preferred treatment option for brainstem metastases due to its provision of adequate local control with low morbidity. Accurate targeting of the tumor may limit damage to the surrounding healthy brain tissue, thereby mitigating the neurological decline (3,6,15). Between 1999 and 2014, >20 studies reported the clinical characteristics, radiation doses and outcomes of brainstem metastases following SRS treatment (Table I). In these reports, the median survival time of patients with brainstem metastases ranged from 4–16.8 months (2–21).

In the present case, the patient was initially diagnosed based on clinical and neuroimaging assessments performed at the Department of Neurosurgery, The Affiliated Hospital of Beihua University. Consistent with the standard treatment of irradiation of brain metastases, the patient underwent GKRS of the brainstem metastasis over 40 days with a total dose of 54.0 Gy. This dose was deemed appropriate based on the study by Maranzano *et al* (22), who reported that the brain tolerated a single course of radiotherapy of 50–60 Gy in 2 Gy daily fractions. However, in the current case, MRI performed three months following GKRS showed an increase in the size of the cystic mass. The patient was therefore referred to the China-Japan Union Hospital of Jilin University for further treatment.

As part of the clinical evaluation, it was critical to determine whether the mass was a metastatic growth or a high-grade glioma. Server *et al* (23) reported that MRS may be utilized to differentiate high-grade glioma from metastases, particularly when the performed in conjunction with measurement of the peritumoral Cho/NAA ratio, which has a high sensitivity (100%). In the current case, the results of these tests indicated the presence of a metastatic carcinoma.

Stereotactic aspiration is a minimally invasive technique. It is widely used to treat brain abscesses (24), and is an important component of the multimodal treatment of cystic craniopharyngiomas (25). Several studies have also applied stereotactic aspiration for the treatment of glial and metastatic brain tumors (26–28). Higuchi *et al* (26) used a procedure in which stereotactic aspiration followed by GKRS on the same day provided good tumor control in 25 patients with cystic metastases. Park *et al* (27) performed stereotactic aspiration, which was followed by GKRS, in 24 patients with cystic metastatic brain tumors. Following treatment, 13 patients (54.2%) had good tumor control, five patients (20.8%) exhibited local tumor progression, and six patients (25.0%) had remote progression. The overall median survival time was 17.8 months, and no cases of brainstem metastasis were identified. To the best of our knowledge, Aung *et al* (29) were the first to report a patient with adenocarcinoma of the right main bronchus that disseminated to the pons, left cerebral peduncle, and liver. In this case, CT-guided stereotactic aspiration of the cystic pontine lesion was performed and a catheter inserted in the cyst cavity was connected to a subgaleal Ommaya reservoir for further aspiration and decompression. Although these procedures alleviated the patient’s clinical symptoms, the patient succumbed to the disease three weeks following the stereotactic surgery, after becoming comatose, with jaundice and ascites. Matsumoto *et al* (30) reported a patient with a cystic metastasis in the midbrain that was successfully treated by brachytherapy following stereotactic biopsy and aspiration of the intratumoral cyst. The authors reported that stereotactic aspiration of cystic lesions provides clinical improvement, and brachytherapy prevents cyst recurrence. In our previous report (31), we treated a brainstem cystic glioma by combining stereotactic aspiration with GKRS, with a favorable outcome. Therefore, we speculated that stereotactic aspiration surgery may improve the effect of GKRS treatment of cystic brainstem glioma.

In the present case, however, the cystic mass progressed and disease control was lost following the initial GKRS. As the results from prior reports and our previous case indicated that stereotactic aspiration of the cystic mass may be an effective treatment, the patient underwent CT-guided stereotactic biopsy, which yielded 2.0 cm^3^ of cystic fluid. Histopathological analysis of the exudate confirmed the origin as primary adenosquamous carcinoma of the lung. Following stereotactic aspiration, GKRS was repeated, reducing the volume of the mass to 1.9 cm^3^. An MRI scan performed five months following the repeat GKRS revealed a controlled mass of 1.2 cm^3^ without substantial radiotherapy-induced edema. However, a second metastatic lesion was detected in the cerebellum of this patient following the second GKRS. The patient survived for 17 months following the first MRI (12 months after the repeat GKRS).

Adenosquamous carcinoma is an uncommon lung cancer, accounting for 0.4–4% of all primary lung cancers, in which ≥10% of the tumor volume contains adenocarcinoma and squamous cell carcinoma components. Adenosquamous carcinomas have a significantly poorer prognosis compared with adenocarcinomas and squamous cell carcinomas. Brain metastasis is common in patients with adenosquamous carcinoma of the lung, however, metastasis to the brainstem from this carcinoma is extremely uncommon (32–35), and the present case is the first in which adenosquamous carcinoma of the lung with metastasis to the brainstem has been reported. Furthermore, the current case is the first to report stereotactic aspiration and GKRS for the treatment of a cystic brainstem metastasis. This treatment provided good local tumor control and the patient survived for 12 months following the procedure. The patient’s survival time of 17 months from initial diagnosis is longer than that of patients reported elsewhere. This indicates that the use of multiple treatment modalities, including stereotactic aspiration combined with GKRS, may extend the survival time of patients with cystic brainstem metastases.

In conclusion, this case suggests that cyst drainage by stereotactic aspiration improves the effects of GKRS in the treatment of cystic brainstem metastasis, by reducing the tumor burden and enhancing local tumor control. To date, there are no evident-based guidelines for the treatment of brainstem metastasis; for the brainstem metastasis with cystic components, multimodality strategy combing stereotactic aspiration with GKRS may be considered. Furthermore, large series of patients with cystic brainstem metastasis should be studied to make the final disgnosis.

## Figures and Tables

**Table I:** Previously published studies of stereotactic radiosurgery for brainstem metastases.

Author (ref)	Year	Patients, n	Lesions, n	Patients with WBRT, n	SRS modality	Median age, years	Median tumor volume, cm^3^	Median tumor margin dose, Gy	Median follow-up, months	Median survival, months	Local control rate,%
Huang *et al* (2)	1999	26	27	24	GKRS	62	1.1	16	9.4	9	95
Shuto *et al* (3)	2003	25	31	9	GKRS	54	2.1	13	5.2	4.9	77.4
Fuentes *et al* (4)	2006	28	28	6	GKRS	57.5	2.1	19	11	12	92
Yen *et al* (5)	2006	53	53	21	GKRS	57.3	2.8	18	9.8	11	86.5
Hussain *et al* (6)	2007	22	25	3	GKRS	60	0.9	16	8.5	8.5	100
Kased *et al* (7)	2008	42	44	24	GKRS	55	0.26	16	6.9	9	77
Lorenzoni *et al* (8)	2009	25	27	17	GKRS	53	0.6	20	10.5	11.1	95
Samblas *et al* (9)	2009	28	30	27	LINAC	52.3	1.86	11.1	NA	16.8	NA
Koyfman *et al* (10)	2010	43	43	34	GKRS	59	0.37	15	5.3	5.8	85
Yoo *et al* (11)	2011	32	NA	NA	GKRS	50	1.5	15.9	12	7.7	87.5
Hatiboglu *et al* (12)	2011	60	60	9	LINAC	61	1	15	5.3	4	76
Valery *et al* (13)	2011	30	43	4	LINAC	57	2.82	13.4	10.4	10	90
Kelly *et al* (14)	2011	24	24	23	LINAC	57	0.2	13	6.6	5.3	78.6
Li *et al* (15)	2012	28	32	0	GKRS	61	0.78	16	NA	9	90.6
Kawabe *et al* (16)	2012	200	222	13	GKRS	64	0.2	18	NA	6	81.8
Lin *et al* (17)	2012	45	48	17	LINAC	59.9	0.4	14	NA	11.6	88
Sengaez *et al* (18)	2013	44	46	29	GKRS	57	0.6	16	NA	8	96
Jung *et al* (19)	2013	32	32	19	GKRS	50	0.711	13	6	5.2	87.5
Kilburn *et al* (20)	2014	44	52	25	GKRS	57	0.134	18	10	6	88
Peterson *et al* (21)	2014	41	NA	19	GKRS	59	0.66	17	NA	4.40	91

WBRT, whole brain radiation therapy; SRS, stereotactic radiosurgery; GKRS, gamma knife radiosurgery; LINAC, linear accelerator; NA, not available.

## Abbreviations

SRS: stereotactic radiosurgery
GKRS: gamma knife radiosurgery
MRI: magnetic resonance imaging
CT: computed tomography
MRS: magnetic resonance spectroscopy
